# Bladder-embedded ectopic intrauterine device with calculus

**DOI:** 10.1515/med-2020-0173

**Published:** 2020-06-08

**Authors:** Bing-Jian Xiong, Guang-Jing Tao, Duo Jiang

**Affiliations:** Department of Urology, Ankang City Central Hospital, Ankang, Shaanxi, 725000, China

**Keywords:** cystectomy, intrauterine device, intrauterine device migration, laparoscopy, surgery, urinary bladder, urinary bladder calculi

## Abstract

The present study aimed to analyze the data of embedded intrauterine device (IUD) in the bladder wall with the additional presence of calculus. This case series study included 11 female patients with partially or completely embedded IUD in the bladder wall. Their median age was 34 (range, 32–39) years. The median duration of IUD placement was 36 (range, 24–60) months. The median duration of symptoms was 9 (range, 3–12) months. Six patients underwent laparoscopy: the operation duration was 129 (range, 114–162) min, blood loss was 15 (range, 10–25) mL, the hospital stay was 4 (range, 4–4.5) days, the visual analog scale (VAS) for pain at 6 h after surgery was 3 (range, 2–6), and the time to removal of the urethral catheter was 7 (range, 7–8) days. Five patients underwent open surgery: the operation duration was 126 (range, 96–192) min, blood loss was 30 (range, 20–50) mL, the hospital stay was 7 (range, 7–15) days, the VAS was 6 (range, 4–9) at 6 h after surgery, and the time to removal of the urethral catheter was 9 (range, 8–17) days. The IUD and bladder stones were successfully removed in all 11 (100%) patients.

## 1 Introduction

Intrauterine device (IUD) insertion is a routine procedure for female contraception and is considered safe and convenient [1,2,3]. The rate of IUD use in the world averages 14.3%, varying from 27% in Asia to 1.8% in Oceania [4]. Some complications may occur, including expulsion (2–10%) [2], pelvic inflammatory disease (2%) [5], pelvic actinomycosis [6], and unplanned pregnancy [2].

Another rare complication is the IUD shifting and embedding itself in the urinary bladder [7]. The occurrence of ectopic IUD is not associated with the shape and duration of the use of the IUD [8]. Possible causes of ectopic IUD have been suggested [9,10], but no formal study was performed on the subject because of the rarity of the cases. First, a weak uterine wall after parturition followed by an early and unskillful insertion may lead to the IUD getting embedded in the uterine wall and possibly further shifting. Second, abnormal morphology and the periodic contraction of the uterus may lead to the IUD’s ectopic shifting. Third, to reduce the side effects of IUD placement, the material and shape of IUDs are continuously optimized and improved, but unsuitable material and shape may lead to chronic incision to the uterine wall during uterine contraction, ultimately leading to the shifting and embedding of the IUD in the posterior wall of the bladder.

So far, this condition has been treated with traditional open cystotomy and IUD removal [11], but it may involve a long incision, bleeding, pain, prolonged immobilization, prolonged hospitalization, and considerable psychological burden. Laparoscopic techniques generally achieve satisfactory curative results for a wide variety of benign and malignant conditions of the bladder [12,13,14], but only a few studies reported laparoscopic cystotomy and IUD removal for translocation (total or partial) of the IUD in the bladder [15,16,17]. Shin et al. [15] reported one patient successfully treated using laparoscopic partial cystectomy. Atakan et al. [16] reported one case operated by suprapubic cystotomy. Jin et al. [17] reported one woman operated using a combination of laparoscopy and air cystoscopy.

Previous studies only report about one or two cases of ectopic IUD embedded in the bladder, but 11 such cases were operated at our institution. Therefore, considering the rarity of the condition and the lack of predefined treatment approach, the aim of the present retrospective study was to analyze the data of all 11 patients who underwent surgery for an IUD embedded in the bladder wall with the additional presence of a calculus and to describe the clinical features and surgical outcomes of this rare condition.

## 2 Methods

### 2.1 Patients

This was a case series of 11 female patients who were diagnosed with partially or completely embedded IUD in the bladder wall with the additional presence of a calculus, and who underwent surgery between January 2008 and December 2017 at the Department of Urology of Ankang City Central Hospital. This study was approved by the ethics committee of our Hospital, with a waiver for individual consent.

The inclusion criteria were as follows: (1) diagnosis of bladder stone caused by ectopic IUD embedded in the bladder and (2) the ectopic IUD was removed by open surgery or laparoscopic surgery. The exclusion criterion was an incomplete medical record of the surgery and the patient’s history. The diagnostic criteria for bladder-embedded IUD were as follows [15,16,17]: (1) abdominal pain, hematuria, and other clinical symptoms consistent with a vesical lesion and (2) ultrasound, CT, and other imaging examinations showed that the IUD was not in the uterine cavity, but partly or completely embedded in the bladder wall.


**Ethics statement**: This study was approved by the ethics committee of Ankang City Central Hospital, with a waiver for individual consent.

### 2.2 Surgical techniques

All interventions were performed by the same senior surgeon, an associate chief physician specialized in laparoscopic and endoscopic minimally invasive techniques.

For traditional open surgery, the preoperative preparation was routinely performed as per any abdominal operation. Spinal anesthesia was used. An 8 cm long incision was made in the middle of the lower abdomen, 3 cm above the symphysis pubis. The abdominal wall was opened layerwise. After the IUD was found at the most severe site of adhesion, the inflammatory thickened adhesion tissues were separated, the posterior wall of the bladder was incised, the IUD was removed, the inflammatory tissues adhering to the IUD were excised, and the bladder was sutured. The closure of the peritoneum and the abdominal cavity was performed. Finally, an F24 pelvic drainage tube was placed near the suture of the pelvic bladder. Catheter drainage of the urine was performed. The urine color was examined, as well as the pelvic drainage volume. The pelvic drainage tube was removed 48–72 h after operation. A urethral catheter was placed and removed 10–12 days after the operation.

For laparoscopy, the supine Trendelenburg position was adopted after tracheal intubation anesthesia. A 3-lumen urinary catheter was inserted into the bladder. An incision of about 1.0 cm was made below the umbilicus. A pneumoperitoneum was established through this incision by needle puncture. A 10 mm trocar was placed at the correct surgical site while making sure that there was no iatrogenic injury to the abdominal organs. The rectus abdominis muscle and anterior superior iliac spine were regarded as the reference points to install three or four trocars. The ectopic IUD was found by using laparoscopic tools such as separating plier, harmonic scalpel, and needle holder, and it was dissociated from the tissues. After filling the bladder with about 300 mL of physiological saline, the IUD and tissues in the posterior wall of the bladder were removed simultaneously. Continuous suture of the bladder wall was performed with 3-0 Vicryl absorbable suture. An extra suture was performed to prevent leakage. A urethral catheter was placed and removed 10–12 days after the operation.

### 2.3 Outcomes

The operation duration, bleeding volume, postoperative hospital stay, visual analog scale (VAS) score for pain (at 6 h after the operation), recovery time, and postoperative complications were extracted from the medical charts. The operation duration was recorded as the time from skin incision to suture closure of the skin. The complications included iatrogenic injury, bleeding, urinary fistula, wound infection, urinary tract infection, and postoperative pain.

The pain was scored on 10 points using a 10 cm long VAS marked with “0” and “10” at each end, with 0 representing no pain and 10 points representing the most unbearable severe pain imaginable. The recovery time was defined as the time between surgery and the removal of the urethral catheter after the restoration of autonomous urination and autonomous activity. Time to discharge was defined as the time between the surgery and removal of the pelvic drainage tube, normal diet, and no need for any special treatment.

All patients were followed routinely over the phone at 3–6 months after the operation. They were routinely inquired about whether they had abdominal pain, incision pain, or abnormal urination. If no complaint was made, no additional follow-up was performed by the medical team, and the patient was asked to consult in case of any symptoms or signs.

### 2.4 Statistical analysis

SPSS 16.0 (SPSS, Chicago, IL, USA) was used for statistical analysis. Only descriptive statistics were used. The continuous variables were presented as median (range). The categorical variables were expressed as numbers and percentages.

## 3 Results

### 3.1 Characteristics of the patients

**Figure 1:**
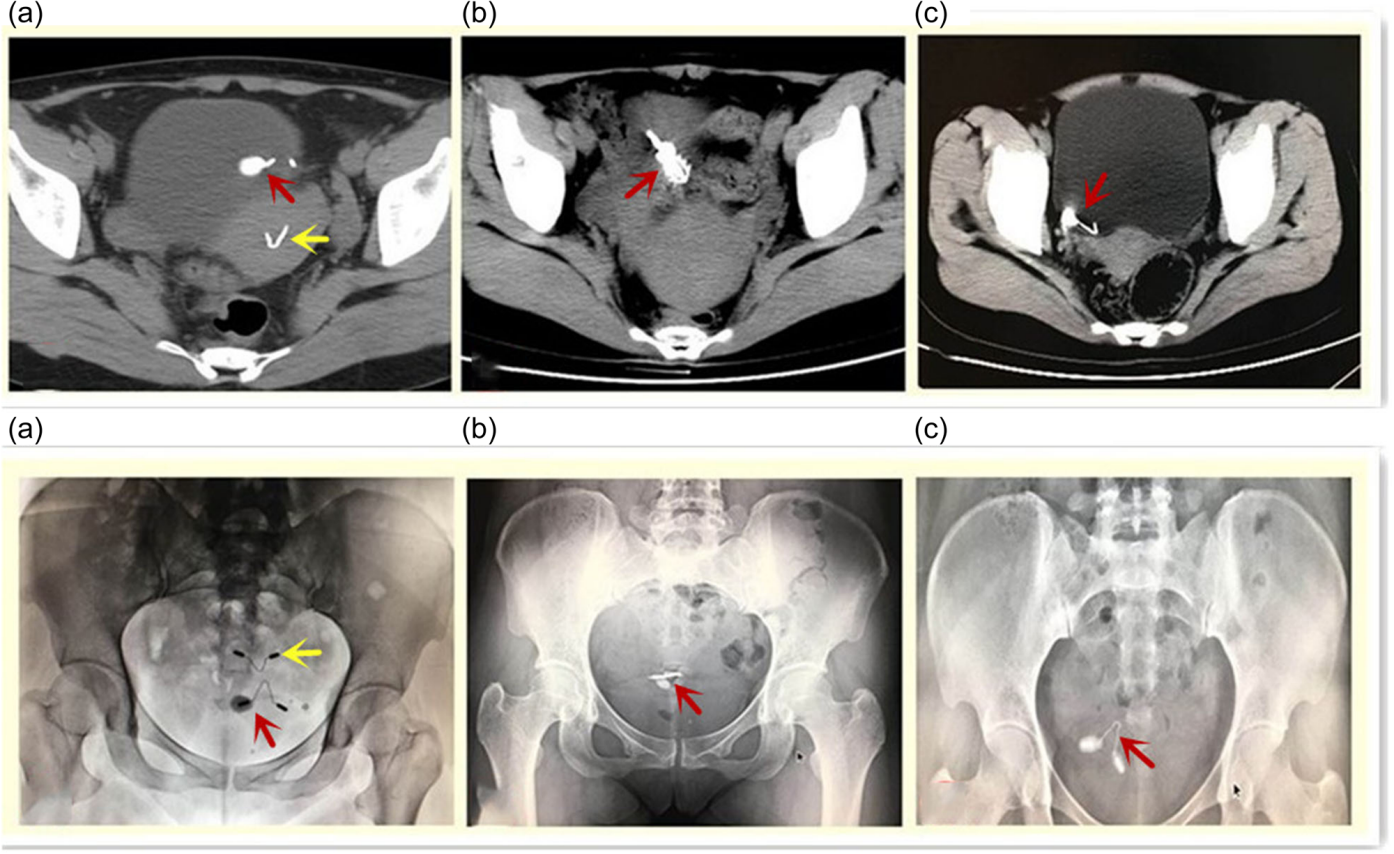
The ectopic IUD was examined by pelvic radiography and computed tomography (CT) and was found to be embedded in the bladder wall and wrapped with a calculus. (a) A 34-year-old patient, gravida 2, para 2 (case #1). The patient got pregnant after IUD insertion. After the induced abortion, a new IUD was inserted. The patient suffered from abdominal pain and hematuria for half a year. Two IUDs can be seen, one is in the uterus (yellow arrow), and the other is embedded in the left posterior wall of the bladder (red arrow). (b) A 33-year-old patient, gravida 2, para 1 (case #2). The patient suffered from abdominal pain and hematuria for 1 year. The ectopic IUD was completely embedded in the posterior wall of the bladder (red arrow). (c) A 34-year-old patient, gravida 2, para 2 (case #5). The patient suffered from intermittent hematuria and urodynia for 9 months. The IUD was completely shifted in the bladder (red arrow).

**Figure 2:**
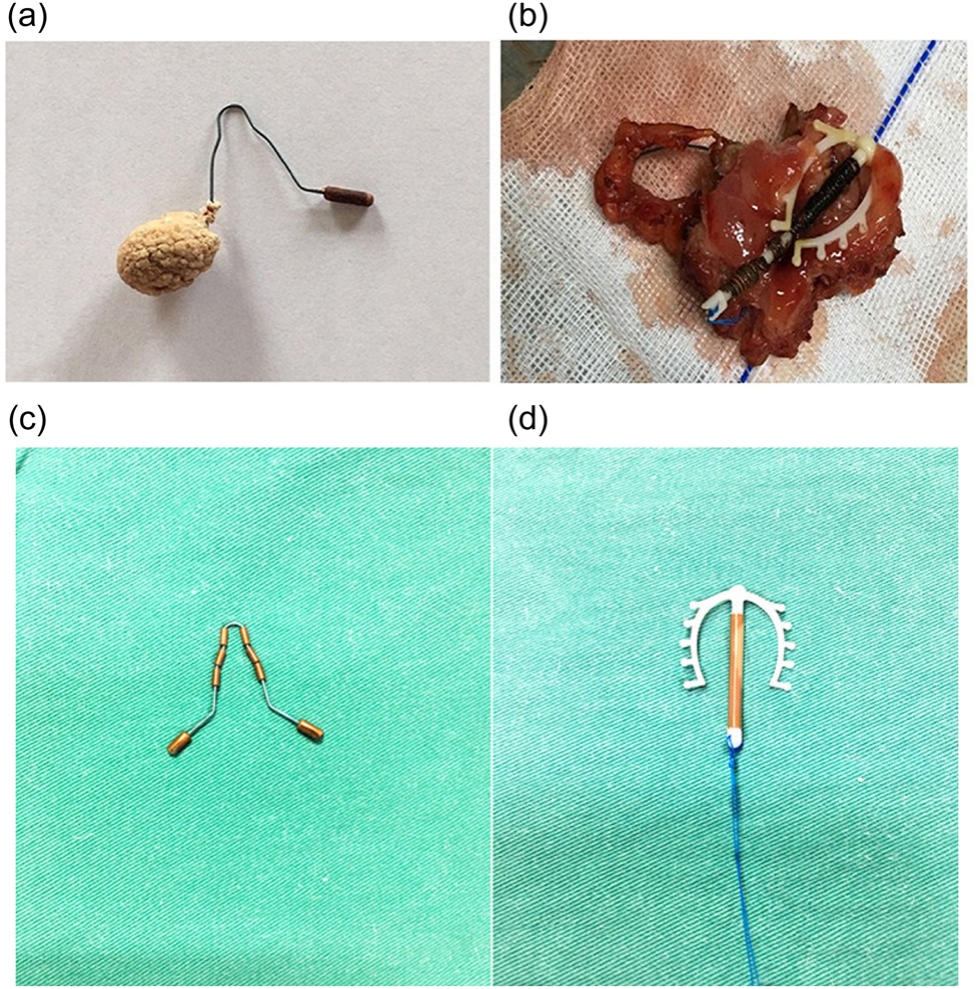
The IUD and associated calculus. (a) The IUD was an MYCu, one end of which was wrapped with the calculus. (b) The IUD was embedded in the bladder wall. (c) Normal appearance of the MYCu IUD. (d) Normal appearance of the Multiload Cu IUD.

During the study period, about 8,000 patients were operated for urological diseases at our hospital, including 11 (0.13%) patients for an IUD that was translocated to the bladder and with an accompanying calculus. Five patients underwent open surgery between January 2008 and December 2012, and six patients underwent laparoscopy between January 2013 and December 2017 due to newer surgical equipment and advances in surgical techniques. The patients were 34 (range, 32–39) years of age. The duration of IUD use was 36 (range, 24–60) months. The duration of symptoms was 9 (range, 3–12) months (Table 1). The median size of the vesical calculi was 1.3 (range, 0.9–1.8) cm, according to imaging (ultrasound, computed tomography, or plain X-ray; Figure 1). No serious complications such as peritonitis or ileus were reported. Surgery was performed successfully in all 11 (100%) patients, and the ectopic IUDs were removed (Figure 2).

**Table 1:** Characteristics of the patients

No	Surgery	Age (years)	Childbearing history	Duration of IUD placement (months)	Symptoms	Duration of symptoms (months)	Imaging diagnosis	Size (cm)	IUD type	Complications
1	Laparoscopy	34	G2P2	24	None	NA	Vesical calculus	1.2	MYCu	None
2	Laparoscopy	33	G2P1	48	Abdominal pain, hematuria	12	Bladder foreign body	1.7	Multiload Cu	None
3	Laparoscopy	32	G1P1	28	Hematuria	6	Bladder foreign body	1.5	MYCu	None
4	Laparoscopy	35	G2P1	36	None	NA	Vesical calculus	1.0	MYCu	None
5	Laparoscopy	34	G2P2	33	Abdominal pain	9	Vesical calculus	1.3	Gynefix	None
6	Laparoscopy	33	G1P1	36	Hematuria	12	Bladder foreign body	1.6	MYCu	None
7	Open	35	G2P2	60	None	NA	Vesical calculus	0.9	Copper t	None
8	Open	33	G2P2	36	Abdominal pain	3	Bladder foreign body	1.6	MYCu	None
9	Open	37	G1P1	39	Hematuria	9	Vesical calculus	1.1	Gynefix	None
10	Open	39	G2P2	48	None	NA	Vesical calculus	1.2	Unknown	Wound infection
11	Open	34	G1P1	36	Abdominal pain	10	Vesical calculus	1.8	Unknown	Wound infection
Total		34 (32, 39)		36 (24, 60)		9 (3, 12)		1.3 (0.9, 1.8)		

G: gravidity, P: parity; IUD: intrauterine device; NA: not applicable.

### 3.2 Characteristics of the surgeries

Six patients underwent laparoscopy: the operation duration was 129 (range, 114–162) min, blood loss was 15 (range, 10–25) mL, the hospital stay was 4 (range, 4–4.5) days, the VAS score for pain at 6 h after surgery was 3 (range, 2–6), and the time to the removal of the urethral catheter was 7 (range, 7–8) days. Five patients underwent open surgery: the operation duration was 126 (range, 96–192) min, blood loss was 30 (range, 20–50) mL, the hospital stay was 7 (range, 7–15) days, the VAS at 6 h was 6 (range, 4–9), and the time to the removal of the urethral catheter was 9 (range, 8–17) days (Table 2).

**Table 2:** Surgical data of the patients

No.	Operation time (min)	Bleeding volume (mL)	Hospital stay (days)	VAS score	Recovery time (days)
1	114	15	4	6	7
2	120	10	4.5	3	7
3	162	25	4	2	8
4	132	15	4	3	7
5	144	17	4	2	7
6	126	12	4	4	7

Total	129 (114, 162)	15 (10, 25)	4 (4, 4.5)	3 (2, 6)	7 (7, 8)
7	126	25	7	4	8
8	108	20	7	8	9
9	96	30	7	9	9
10	174	38	15	6	17
11	192	50	10	5	14

Total	126 (96, 192)	30 (20, 50)	7 (7, 15)	6 (4, 9)	9 (8, 17)

VAS: visual analog scale.

## 4 Discussion

IUD is a common contraceptive method for women of childbearing age. It is safe, effective, and easy to insert. Nevertheless, some complications may occur and include migration and embedding into the urinary bladder wall [7]. In this study, 11 patients with bladder stones caused by an ectopic IUD embedded in the bladder wall were retrospectively analyzed. Among them, six patients underwent laparoscopic surgery and five patients underwent traditional open surgery. Bladder-embedded ectopic IUD accompanied with calculus is a rare condition, but the study suggests that it can be treated successfully with either laparoscopy or open surgery.

The clinical symptoms of ectopic IUD may vary among patients but may include colporrhagia, hypogastralgia, abdominal pain, increased urinary frequency, urinary urgency, urodynia, and hematuria, depending upon the location of the IUD, and some patients can have no symptoms after IUD migration [18,19]. The patients included in the present study showed obvious lower urinary tract syndrome characterized by urgent urination, dysuria, and hematuria. Imaging (including ultrasound, computed tomography, and plain X-ray) and cystoscopy provide strong diagnostic support and also play a key role in selecting the surgical methods and approaches [20]. In this study, each IUD was deeply embedded in the bladder wall and could not be removed by hysteroscopy or cystoscopy. The largest vesical calculus was 1.8 cm. Hence, the patients had to undergo proper surgery. In the present study, both open surgery and laparoscopic surgery were successful in removing the ectopic IUD without major complications. Laparoscopy is usually associated with smaller scars than open surgery, which is more in line with the aesthetic needs of younger women, and with smaller blood loss, less pain, and shorter hospital stay as supported by a previous study [21]. Under the premise that both laparoscopic and open surgery can successfully remove the ectopic IUD and bladder stone, laparoscopy should be preferred, considering its advantages such as smaller incision, less blood loss, less pain, and shorter hospital stay. Nevertheless, if the removal of the IUD and bladder stone fails under laparoscopy, open surgery is needed. For open surgery of the lower abdomen, spinal anesthesia is appropriate. General anesthesia can be performed as well, but it is not necessary and is more expensive. Therefore, patients underwent open surgery in this study under spinal anesthesia. Laparoscopy requires the establishment of a pneumoperitoneum, so all the patients were operated under general anesthesia.

This study included six patients who underwent laparoscopy and suggested that this procedure can be used successfully for the treatment of an IUD embedded within the bladder wall with the additional presence of a calculus. Only some case reports presented laparoscopic surgery for ectopic IUD [15,16,17,22,23,24,25,26]. Shin et al. [15], Jin et al. [17], Rahnemai-Azar et al. [23], Santos et al. [24], Liu et al. [22], Yahsi et al. [25], and Kurdoglu et al. [26] each reported one patient successfully operated using laparoscopic partial cystectomy or open surgery. Atakan et al. [16] reported one case operated by suprapubic cystotomy. These reports suggest that both open and laparoscopic surgeries are appropriate for ectopic IUD embedded in the bladder wall. Nevertheless, other methods are also available and could be explored. Indeed, Sano et al. [27] reported one case operated with endoscopy and laser fragmentation of the calculus surrounding the IUD. Chai et al. [9] used cystoscopy to remove the IUD, while Zhang et al. [28] used a combination of various endoscopes. IUDs are usually implanted at childbearing age and are recommended to be removed after menopause. Nevertheless, some patients do not have their IUD removed after menopause, for various reasons, and IUD displacement can happen at an old age. If there is no symptom, we recommend conservative treatments. If the patients have hematuria, abdominal pain, or recurrent urinary tract infections, they should choose surgical treatment. Since surgeries are always associated with some risks such as adhesion or iatrogenic injury, it has been suggested that patients without symptoms or patients with high surgical risk factors, especially elderly patients, might be more suitable for conservative treatment [18]. The traditional method of suturing the bladder is to suture the whole layer of the incision first and then suture the sarcoplasmic layer to prevent leakage. Laparoscopic suturing of the bladder is more difficult. At our center, we use a continuous suture from the left side of the incision, with the assistant holding the end of the suture to maintain the tension before knotting. Then, we reverse to suture from the right side of the incision using the same method. Using this method, one suture is enough, as per our observation. Nevertheless, this method has not been reported before and should be examined more closely. In addition, new products such as barbed sutures, which were not available at our center during the study period, might provide satisfactory results [29].

This study has limitations. This was a retrospective study of all patients IUD getting embedded in the bladder wall with the additional presence of a calculus treated at one hospital. The number of patients was too small to be able to compare open versus laparoscopic surgeries. Due to the rarity of the condition, the wide range of IUD available on the market, and the conditions specific to each patient, the selection of laparoscopy versus open surgery should be tailored to each specific case. Finally, based on our observation, a calculus forms when the IUD comes in contact with urine. We did observe even rarer cases of ectopic IUD without contemporary calculus, but they were not included, as per the inclusion criteria, and no data were collected. Long-term studies with larger sample sizes are needed to confirm the results.

An ectopic IUD getting embedded in the bladder wall with the additional presence of a calculus is rare. Both open surgery and laparoscopy can be used to remove the ectopic IUD and bladder stone successfully.
