# Erratum to “Bladder-embedded ectopic intrauterine device with calculus”

**DOI:** 10.1515/med-2023-1000

**Published:** 2024-02-14

**Authors:** Bing-Jian Xiong, Guang-Jing Tao, Duo Jiang

**Affiliations:** Department of Urology, Ankang City Central Hospital, Ankang, Shaanxi, 725000, China

In the published article Xiong B J, Tao G J, Jiang D. Bladder-embedded ectopic intrauterine device with calculus. Open Med. (Wars) 2020 15(1): 501–507. doi: 10.1515/med-2020-0173. PMID: 33336004; PMCID: PMC7712230, due to proofreading errors and misidentification of IUD, IUD types of case 5 and case 9 were incorrectly written as GyneFix in Table 1. Authors requested to modify the IUD type for both cases, with case 5 being changed to MYCu and case 9 being rendered unrecognizable. The changes do not affect the results and conclusions of the study.

**Table 1:** Characteristics of the patients

No	Surgery	Age (years)	Childbearing history	Duration of IUD placement (months)	Symptoms	Duration of symptoms (months)	Imaging diagnosis	Size (cm)	IUD type	Complications
1	Laparoscopy	34	G2P2	24	None	NA	Vesical calculus	1.2	MYCu	None
2	Laparoscopy	33	G2P1	48	Abdominal pain, hematuria	12	Bladder foreign body	1.7	Multiload Cu	None
3	Laparoscopy	32	G1P1	28	Hematuria	6	Bladder foreign body	1.5	MYCu	None
4	Laparoscopy	35	G2P1	36	None	NA	Vesical calculus	1.0	MYCu	None
5	Laparoscopy	34	G2P2	33	Abdominal pain	9	Vesical calculus	1.3	MYCu	None
6	Laparoscopy	33	G1P1	36	Hematuria	12	Bladder foreign body	1.6	MYCu	None
7	Open	35	G2P2	60	None	NA	Vesical calculus	0.9	Copper t	None
8	Open	33	G2P2	36	Abdominal pain	3	Bladder foreign body	1.6	MYCu	None
9	Open	37	G1P1	39	Hematuria	9	Vesical calculus	1.1	Unidentifiable	None
10	Open	39	G2P2	48	None	NA	Vesical calculus	1.2	Unknown	Wound infection
11	Open	34	G1P1	36	Abdominal pain	10	Vesical calculus	1.8	Unknown	Wound infection
Total		34 (32, 39)		36 (24, 60)		9 (3, 12)		1.3 (0.9, 1.8)		

G: gravidity, P: parity; IUD: intrauterine device; NA: not applicable.

